# The use of national administrative data to describe the spatial distribution of in-hospital mortality following stroke in France, 2008–2011

**DOI:** 10.1186/s12942-015-0028-2

**Published:** 2016-01-11

**Authors:** Adrien Roussot, Jonathan Cottenet, Maryse Gadreau, Maurice Giroud, Yannick Béjot, Catherine Quantin

**Affiliations:** Service de Biostatistique et d’Informatique Médicale (DIM), CHRU Dijon, Dijon, 21000 France; Université de Bourgogne, Dijon, 21000 France; Laboratoire d’Economie de Dijon, Université de Bourgogne, UMR 6307 CNRS, INSERM U1200, Dijon, France; Registre des AVC dijonnais, EA4184, CHRU, Univ de Bourgogne, Dijon, France; INSERM, CIC 1432, Dijon, France; Clinical Epidemiology/Clinical Trials Unit, Clinical Investigation Center, Dijon University Hospital, Dijon, France; Biostatistics, Biomathematics, Pharmacoepidemiology and Infectious Diseases (B2PHI), Univ. Bourgogne Franche-Comté, Inserm UMR 1181, Dijon, 21000 France

**Keywords:** PMSI, Stroke, Geography, In-hospital mortality

## Abstract

**Background:**

In the context of implementing the National Stroke Plan in France, a spatial approach was used to measure inequalities in this disease. Using the national PMSI-MCO databases, we analyzed the in-hospital prevalence of stroke and established a map of in-hospital mortality rates with regard to the socio-demographic structure of the country.

**Methods:**

The principal characteristics of patients identified according to ICD10 codes relative to stroke (in accordance with earlier validation work) were studied. A map of standardized mortality rates at the level of PMSI geographic codes was established. An exploratory analysis (principal component analysis followed by ascending hierarchical classification) using INSEE socio-economic data and mortality rates was also carried out to identify different area profiles.

**Results:**

Between 2008 and 2011, the number of stroke patients increased by 3.85 %, notably for ischemic stroke in the 36–55 years age group (60 % of men). Over the same period, in-hospital mortality fell, and the map of standardized rates illustrated the diagonal of high mortality extending from the north-east to the south-west of the country. The most severely affected areas were also those with the least favorable socio-professional indicators.

**Conclusions:**

The PMSI-MCO database is a major source of data on the health status of the population. It can be used for the area-by-area observation of the performance of certain healthcare indicators, such as in-hospital mortality, or to follow the implementation of the National Stroke Plan. Our study showed the interplay between social and demographic factors and stroke-related in-hospital mortality. The map derived from the results of the exploratory analysis illustrated a variety of areas where social difficulties, aging and high mortality seemed to meet. The study raises questions about access to neuro-vascular care in isolated areas and in those in demographic decline. Telemedicine appears to be the solution favored by decision makers. The aging of the population managed for stroke must not mask the growing incidence in younger people, which raises questions about the development of classical (smoking, hypertension) or new (drug abuse) risk factors.

## Background

The management of stroke is becoming an increasingly heavy burden worldwide for both developed and developing countries. Though the incidence rate of the disease in rich countries has fallen over the last 20 years, prevalence has increased as the quality of stroke management has improved [1, 2]. It is becoming necessary to evaluate responses provided by healthcare systems and then to implement public healthcare actions to cope with the burden of this disease in both the acute and chronic phase.

Since the year 2000, France has been engaged in a battle against stroke, and since 2010, all of the actions in the management of stroke patients have been incorporated in a “2010–2014 National Stroke Action Plan” [3]. This plan, which was based on a comprehensive ‘inventory’ requested by the Ministry of Health and which associated researchers, the relevant learned societies and representatives of patients [4], emphasizes the need for a territory-wide stroke-care network. At the regional level, emergency stroke management is centered on stroke units and better coordination between these establishments and follow-up care and rehabilitation departments in the downstream management of stroke.

The national strategy in the fight against stroke also involves the development of epidemiological research in stroke, notably through the increased exploitation of existing information systems: PMSI-MCO (Programme de médicalisation des systèmes d’information) and SNIIR-AM (Système national inter-régime de l’assurance maladie) (action 8 of the Plan). Indeed, a number of studies have been conducted thanks to the availability and long-standing nature of medico-administrative databases. The PMSI-MCO in particular is a precious source of information for studies in epidemiology [5] or healthcare economics [6]. Indeed, the PMSI is the main source of French hospitalisation data. For each patient, it gathers all of the hospital stays during the year and allows patients to be followed over time thanks to a unique data linkage number.

Earlier studies on the incidence and prevalence of stroke conducted using the PMSI-MCO showed that the demographics of the population managed for stroke have changed over the last 20 years. This situation is probably related to the greater efficacy of prevention messages and the earlier involvement of the healthcare system. A study published in 2012 [7] underlined the interest of the PMSI-MCO to elaborate and analyze hospital and follow-up care trajectories. The study highlighted the growing importance of stroke units between 2007 and 2009 and concluded that the overall management of stroke was more effective, with a reduction of the in-hospitality mortality.

This work is a continuation of these observational studies on the results of care in stroke patients, in the context of the deployment of the National Action Plan. In this context, we can make the hypothesis that this decrease may be observable for a larger period and that its spatial distribution in France follows an heterogenic organization. We studied the number of stroke victims at the national level in France between 2008 and 2011 using PMSI-MCO medico-administrative databases, as well as the evolution of the principal characteristics of this population, notably with regard to in-hospital mortality. The analysis presents a map of in-hospital mortality, as well as an exploratory analysis of the territorial distribution of this mortality with regard to demographic and socio-economic data.

The aim of this analysis was to describe the spatial distribution of in-hospital mortality related to the socio-economic characteristics of the patients’ places of residence.

## Methods

### Source of data and selection criteria

Given the lack of long-term data in the national PMSI databases and the absence of accurate coding to distinguish between first-ever and recurrent stroke, this study analyses the characteristics of patients hospitalized for stroke rather than the in-hospital incidence.

The algorithm used was the same as that used for other French studies conducted using the national PMSI database. All of the hospital stays for patients presenting with a diagnosis of stroke recorded in the national PMSI-MCO databases from 2008 to 2011, whatever their age, were included. The hospital stays were selected according to the principal diagnosis (PD) according to the following ICD10 codes:

- I60, I61, I62.9 for hemorrhagic stroke.
- I63, I64, G46 for ischemic stroke.

In our selection process, we chose to keep patients with a “transfer” admission, that is to say patients who stayed first in one establishment and then moved to a second establishment with a PD of stroke. In these cases of transfer, only the second stay with a diagnosis of stroke was taken into account for the analysis. All hospital stays ending with in-hospital death (discharge code = 9) were included, even when the hospital stay was 0 days. For deceased patients who had accumulated several hospital stays after a stroke, their death was identified in a period of 30 days following the first hospital stay for stroke. For patients who were transferred during their management, the complete duration of the hospital stay was taken into account.

Selection was limited to geographical codes for France. PMSI geographic codes indicate where patients live; in France these places correspond to zip codes. Hospital stays presenting coding errors shown by a return code or an error in the DRG (Diagnosis Related Group) code were removed.

### Calculation of standardized in-hospital mortality rates

The mortality rate included all of the in-hospital deaths recorded during the period of the study (2008–2011). It was standardized for age according to the direct method, by using as the reference INSEE (Institut National de la Statistique et des Etudes Economiques: French national census institute data of the 2009 population census at the level of the PMSI geographic code for the place of residence. These population data were obtained by aggregation of the census data available for each town using the post code and the corresponding PMSI geographic code established by the ATIH (Agence Technique de l’Information Hospitalière: Technical agency for hospital information) in 2010. Mortality rates are expressed for 100,000 inhabitants. The exploratory analysis is based on commune-by-commune INSEE data for Employed persons—active Population from the 2011 census and aggregated according to the post code and PMSI geographic code.

### Exploratory analysis: typology of the territories according to their socio-economic and demographic structure related to in-hospital mortality

- The exploratory analysis consisted of a principal components analysis (PCA) followed by an ascending hierarchical classification (AHC) using Ward’s step method bearing on several variables of interest at the level of the 5672 PMSI geographic codes of their residence in France. The ascending hierarchical classification (AHC) consisted in gradually aggregating the geographic codes according to their resemblance, which allowed us to predict the cluster of an individual according to the values taken by the predictive variables: Proportion of each occupational category in the active population
- Proportion of unemployed people in the active population
- Proportion of retired or pre-retired people aged 15–64 in the active population
- Proportion of the whole population aged less than 20 years
- Proportion of the whole population aged from 20 to 59 years
- Proportion of the whole population aged more than 60 years
- Standardized rate of in-hospital mortality due to stroke for 100,000 inhabitants

We kept the first three factorial axes from the PCA for the AHC. These allowed us to synthesize 69.2 % of the information. All of the variables selected in the AHC were discriminating for the description and the construction of the different clusters of PMSI geographic areas (cf. Table 5). Only the managers or professional occupations category (SPC n°3) was not retained to describe cluster 4 (p > 0.05). The typology from the AHC was based on 5 classes of affectation for the geographic codes. This apportionment was obtained after interpretation of the associated dendogram, from the cubic clustering criterion (measures the quality of the cluster) and from the semi-partial R^2^ (measures the loss of interclass inertia). The results obtained from the classification are presented in Tables 5 and 6, which present the means of each variable within the clusters, as well as their discriminating power (p < 0.05) for the construction of each cluster. The cartographic results concerning the classification of geographic codes of the place of residence are shown in Fig. 5. For each variable, the legend presents the mean deviation, calculated overall for the geographic codes of the place of residence. The bars on the right of the vertical line represent a positive delta, while those on the left represent a negative delta. The wider the bar, the greater the mean deviation (positive or negative) is.

The description of each cluster can be determined in a complementary manner from Fig. 5, Tables 5 and 6.

SAS 9.3 software was used for all of the analyses. GIS (Geographic Information System) MapInfo 11.0 was used for the cartography.

## Results

### Hospitalizations for AVC: characteristics of the study population

In France, the number of people hospitalized for stroke increased by 3.9 %, from 96,695 to 100,420 between 2008 and 2011 (c.f. Table 1). There was, however, a slight fall in the number of patients between 2010 and 2011 (fall of 1295 cases). In detail, the greatest increase concerned ischemic stroke (+3.9 %); the number of patients hospitalized for hemorrhagic stroke increased by 3.3 %.

**Table 1:** Global description of strokes

Strokes	2008		2009		2010		2011		Total		χ^2^	Somers’d
	n	%	n	%	n	%	n	%	n	%	p	p
All strokes	97,574		99,398		102,593		101,237		400,802			
Hemorrhagic	22,327	22.9	22,618	22.7	23,426	22.8	23,056	22.8	91,427	22.8	NS	NS
Ischemic	75,247	77.1	76,780	77.3	79,167	77.2	78,181	77.2	309,375	77.2		
Age												
All patients												
≤35	2476	2.5	2491	2.5	2586	2.5	2310	2.3	9863	2.5	0.0005	0.0106
36–55	11,464	11.8	11,395	11.5	12,017	11.7	11,706	11.6	46,582	11.6		
>55	83,634	85.7	85,512	86.0	87,990	85.8	87,221	86.2	344,357	85.9		
Male												
≤35	1220	2.5	1202	2.5	1332	2.6	1127	2.3	4881	2.5	0.0058	NS
36–55	6786	14.1	6728	13.8	7069	14.0	7044	14.1	27,627	14.0		
>55	40,255	83.4	40,867	83.8	42,061	83.4	41,644	83.6	164,827	83.5		
Female												
≤35	1256	2.6	1289	2.6	1254	2.4	1183	2.3	4982	2.5	0.0074	0.0013
36–55	4678	9.5	4667	9.2	4948	9.5	4662	9.1	18,955	9.3		
>55	43,379	88.0	44,645	88.2	45,929	88.1	45,577	88.6	179,530	88.2		
Sex												
Male	48,261	49.5	48,797	49.1	50,462	49.2	49,815	49.2	197,335	49.2	NS	NS
Female	49,313	50.5	50,601	50.9	52,131	50.8	51,422	50.8	203,467	50.8		
In-hospital death (in 30 days)												
All patients	16,178	16.6	16,234	16.3	16,700	16.3	16,249	16.1	65,361	16.3	0.0160	0.0009
% age-sexe-adjusted		16.6		16.2		16.2		15.9				
Male	7389	15.3	7430	15.2	7554	15.0	7285	14.6	29,658	15.0	0.0116	0.0006
% age-adjusted		15.3		15.1		14.9		14.6				
≤35	86	7.1	77	6.4	83	6.2	63	5.6	309	6.3	NS	0.0765
36–55	547	8.1	561	8.3	577	8.2	550	7.8	2235	8.1	NS	NS
>55	6756	16.8	6792	16.6	6894	16.4	6672	16.0	27,114	16.5	0.0206	0.0010
Female	8789	17.8	8804	17.4	9146	17.5	8964	17.4	35,703	17.6	NS	NS
% age-adjusted		17.8		17.4		17.6		17.4				
≤35	62	4.9	49	3.8	51	4.1	46	3.9	208	4.2	NS	NS
36–55	416	8.9	407	8.7	374	7.6	386	8.3	1583	8.4	NS	0.0482
>55	8311	19.2	8348	18.7	8721	19.0	8532	18.7	33,912	18.9	NS	NS

From 2008 to 2011, 97,574 people were hospitalized for stroke in France, 22,327 (22.8 %) presented hemorrhagic stroke and 75,247 (77.2 %) ischemic stroke. The distribution according to the year was stable (Table 1).

More women than men were hospitalized for stroke [203,467 (50.8 %) vs. 197,335 (49.2 %) for the whole period] and whatever the year. The differences between the years were not significant (Table 1). The percentage of women hospitalized for hemorrhagic stroke (51.2 %—Table 2) was greater than that for ischemic stroke (50.6 %—Table 3). The difference was significant (*p* = 0.0067). In addition, for hemorrhagic stroke (Table 2), the proportion of women hospitalized increased between 2008 (50.7 %) and 2011 (51.6 %), the difference was significant (*Somers’d* = 0.0452).

**Table 2:** Description of hemorrhagic strokes

Hemorrhagic strokes	2008		2009		2010		2011		Total		χ^2^	Somers’d
	n	%	n	%	n	%	n	%	n	%	p	p
All patients	22,327		22,618		23,426		23,056		91,427			
Age												
All patients												
≤35	1205	5.4	1120	5.0	1169	5.0	1021	4.4	4515	4.9	<10^−3^	<10^−3^
36–55	4093	18.3	3946	17.5	4026	17.2	3935	17.1	16,000	17.5		
>55	17,029	76.3	17,552	77.6	18,231	77.8	18,100	78.5	70,912	77.6		
Male												
≤35	663	6.0	599	5.4	676	5.9	551	4.9	2489	5.6	0.0007	0.0012
36–55	2152	19.6	2106	19.0	2077	18.2	2105	18.9	8440	18.9		
>55	8192	74.4	8365	75.6	8673	75.9	8495	76.2	33,725	75.5		
Female												
≤35	542	4.8	521	4.5	493	4.1	470	4.0	2026	4.3	<10^−3^	<10^−3^
36–55	1941	17.2	1840	15.9	1949	16.2	1830	15.4	7560	16.2		
>55	8837	78.1	9187	79.6	9558	79.7	9605	80.7	37,187	79.5		
Sex												
Male	11,007	49.3	11,070	48.9	11,426	48.8	11,151	48.4	44,654	48.8	NS	0.0452
Female	11,320	50.7	11,548	51.1	12,000	51.2	11,905	51.6	46,773	51.2		
In-hospital death (in 30 days)												
All patients	7153	32.0	7367	32.6	7469	31.9	7438	32.3	29,427	32.2	NS	NS
% age-sexe-adjusted		32.0		32.3		31.6		31.7				
Male	3450	31.3	3511	31.7	3657	32.0	3500	31.4	14,118	31.6	NS	NS
% age-adjusted		31.3		31.5		31.8		31.0				
≤35	72	10.9	58	9.7	68	10.1	58	10.5	256	10.3	NS	NS
36–55	379	17.6	407	19.3	422	20.3	399	19.0	1607	19.0	NS	NS
>55	2999	36.6	3046	36.4	3167	36.5	3043	35.8	12,255	36.3	NS	NS
Female	3703	32.7	3856	33.4	3812	31.8	3938	33.1	15,309	32.7	0.0458	NS
% age-adjusted		32.7		33.1		31.4		32.5				
≤35	47	8.7	44	8.5	39	7.9	39	8.3	169	8.3	NS	NS
36–55	332	17.1	324	17.6	283	14.5	310	16.9	1249	16.5	0.0471	NS
>55	3324	37.6	3488	38.0	3490	36.5	3589	37.4	13,891	37.4	NS	NS

**Table 3:** Description of ischemic strokes

Ischemic strokes	2008		2009		2010		2011		Total		χ^2^	Somers’d
	n	%	n	%	n	%	n	%	n	%	p	p
All patients	75,247		76,780		79,167		78,181		309,375			
Age												
All patients												
≤35	1271	1.7	1371	1.8	1417	1.8	1289	1.7	5348	1.7	0.0235	NS
36–55	7371	9.8	7449	9.7	7991	10.1	7771	9.9	30,582	9.9		
>55	66,605	88.5	67,960	88.5	69,759	88.1	69,121	88.4	273,445	88.4		
Male												
≤35	557	1.5	603	1.6	656	1.7	576	1.5	2392	1.6	0.0347	0.0462
36–55	4634	12.4	4622	12.3	4992	12.8	4939	12.8	19,187	12.6		
>55	32,063	86.1	32,502	86.2	33,388	85.5	33,149	85.7	131,102	85.9		
Female												
≤35	714	1.9	768	2.0	761	1.9	713	1.8	2956	1.9	NS	NS
36–55	2737	7.2	2827	7.2	2999	7.5	2832	7.2	11,395	7.3		
>55	34,542	90.9	35,458	90.8	36,371	90.6	35,972	91.0	142,343	90.8		
Sex												
Male	37,254	49.5	37,727	49.1	39,036	49.3	38,664	49.4	152,681	49.4	NS	NS
Female	37,993	50.5	39,053	50.9	40,131	50.7	39,517	50.6	156,694	50.6		
In-hospital death (in 30 days)												
All patients	9025	12.0	8867	11.6	9231	11.7	8811	11.3	35,934	11.6	0.0002	<10^−3^
% age-sexe-adjusted		12.0		11.5		11.7		11.3				
Male	3939	10.6	3919	10.4	3897	10.0	3785	9.8	15,540	10.2	0.0010	<10^−3^
% age-adjusted		10.6		10.4		10.0		9.8				
≤35	14	2.5	19	3.2	15	2.3	5	0.9	53	2.2	0.0574	0.0088
36–55	168	3.6	154	3.3	155	3.1	151	3.1	628	3.3	NS	0.0493
>55	3757	11.7	3746	11.5	3727	11.2	3629	11.0	14,859	11.3	0.0082	0.0003
Female	5086	13.4	4948	12.7	5334	13.3	5026	12.7	20,394	13.0	0.0023	0.0380
% age-adjusted		13.4		12.7		13.3		12.7				
≤35	15	2.1	5	0.7	12	1.6	7	1.0	39	1.3	NS	NS
36–55	84	3.1	83	2.9	91	3.0	76	2.7	334	2.9	NS	NS
>55	4987	14.4	4860	13.7	5231	14.4	4943	13.7	20,021	14.1	0.0031	0.0466

For all strokes (Table 1), we found an increase in the proportion of people older than 55 hospitalized for stroke from 85.7 % in 2008 to 86.2 % in 2011. The difference was significant *(Somers’d* = 0.0106*)*. This significant increase occurred in women (88.0 % in 2008 vs 88.6 % in 2011: *Somers’d* = 0.0013) but not in men (Table 1). This global trend was related (Table 2) to the substantial increase in the proportion of patients older than 55 years hospitalized for hemorrhagic stroke (76.3 % in 2008 vs 78.5 % in 2011: *Somers’d* < 10^−*3*^). The trend was observed in both men (*Somers’d* = 0.0012) and women (*Somers’d* < 10^−3^*)*. This trend was not found for ischemic strokes overall (Table 3) (*Somers’d non*-*significant*) even though significant differences were found in the distribution of patients by age group (*p* = 0.0235) in the different years. However, the structure by age and sex showed an increase in the proportion of men aged 36–55 years (12.4 % in 2008 vs. 12.8 % in 2011) and a fall in the proportion of those aged more than 55 years (86.1 % in 2008 vs. 85.7 % in 2011: *Somers’d* = 0.0462). We found no statistically significant evolution in the structure according to age group in women.

In contrast (Table 2) the proportion of men aged 36–55 years who presented hemorrhagic stroke fell during the study period (19.6 % in 2008 vs. 18.9 % in 2011) whereas that in patients older than 55 years increased (74.4 % in 2008 vs. 76.2 % in 2011); the differences were significant (*Somers’d* = 0.0012). The same significant trends were observed in women (Table 2) with a decrease in the proportion of women aged less than 36 years (4.8 % in 2008 vs. 4.0 % in 2011) and in those aged 36–55 years (17.2 % in 2008 vs. 15.4 % in 2011) as well as an increase in those aged more than 55 years (78.1 % in 2008 vs. 80.7 % in 2011). These differences were significant *(Somers’d* < 10^−3^*).*

**Fig. 1:**
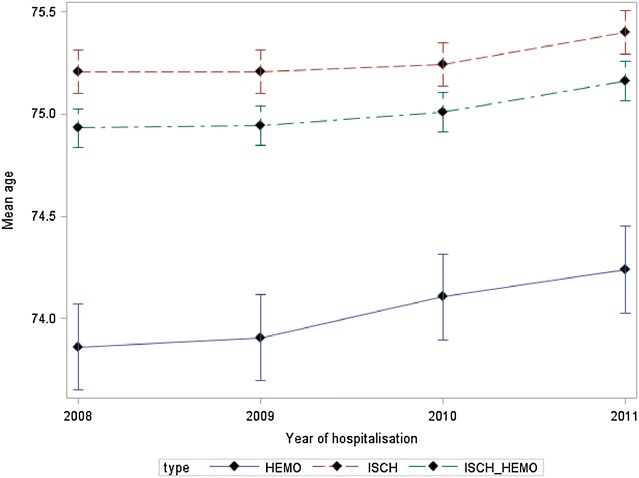
Mean age, male over 55 years old

**Fig. 2:**
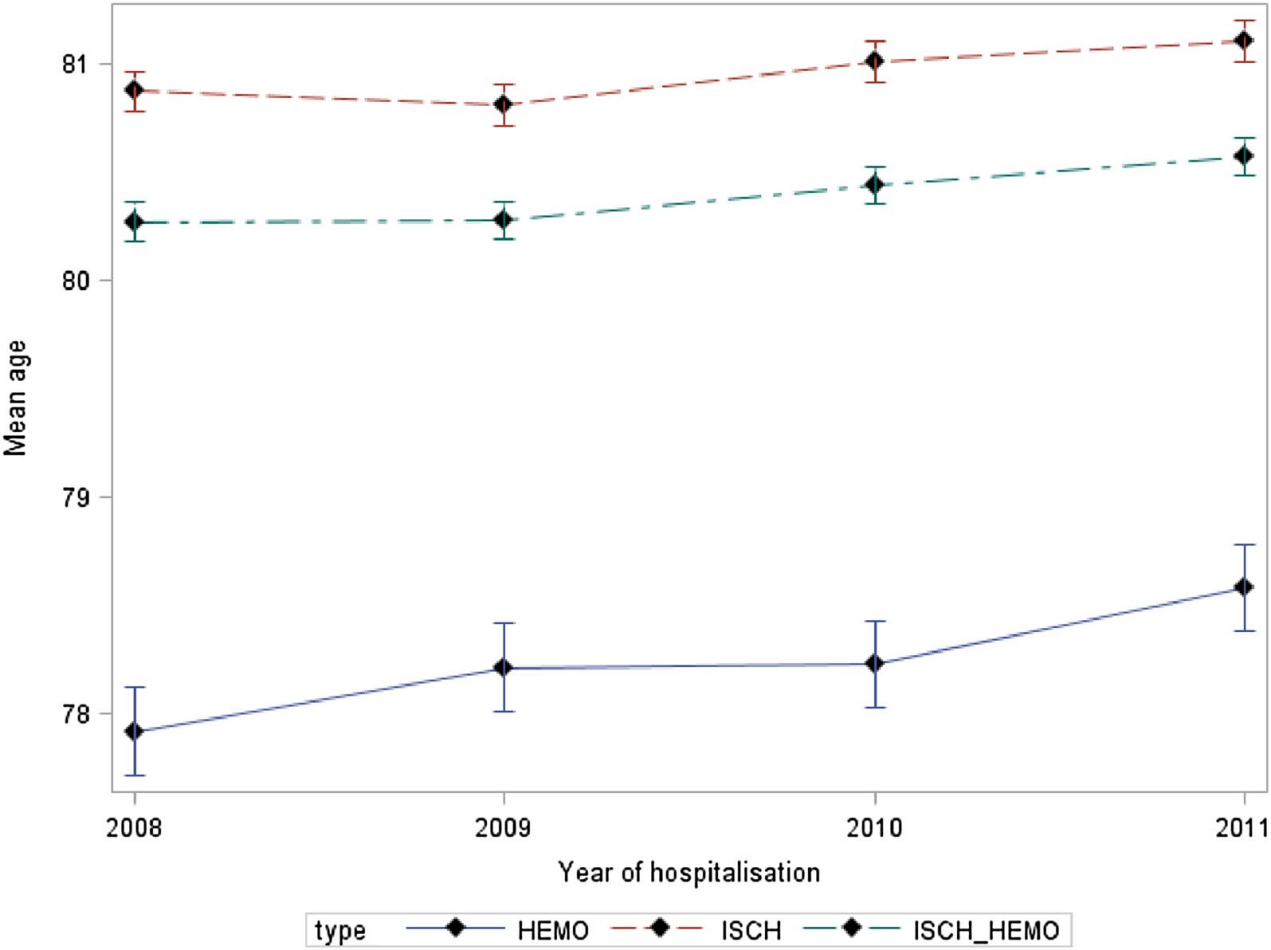
Mean age, female over 55 years old

Whatever the type of stroke and year, the mean age of women in the group aged more than 55 years was higher than that in men: (80.39 years [80.35–80.44] vs. 75.01 years [74.97–75.06] p < 10^−3^) and this whatever the type of stroke: hemorrhagic : 78.24 years [78.14–78.35] vs. 70.03 years [73.93–74.14] (p < 10^−3^); ischemic: 80.96 years [80.91–81.00] vs. 75.27 years [75.21–75.32] (p < 10^−3^). Moreover, for this same age group, we found an increase in the mean age from 2008 to 2011 whatever the sex and type of stroke (Figs. 1, 2).

### In-hospital mortality

For strokes overall (Table 1), we found a significant fall in the raw in-hospital mortality rate, which fell from 16.6 % in 2008 to 16.1 % in 2011, and in the standardized mortality rate (16.6 vs. 15.9 %). The difference was significant (*Somers’d* = 0.0009). This fall in in-hospital mortality was especially due to the fall in the lethality of ischemic stroke (12.0 % in 2008 vs. 11.3 % in 2011; *Somers’d* < 10^−3^) (Table 3), while in-hospital mortality in patients with hemorrhagic stroke (Table 2) remained stable and high (32.0 % in 2008 vs. 32.3 % in 2011; *Somers’d NS)*.

For strokes overall (Table 1), the decrease in the raw and standardized mortality rates was only significant in men (*Somers’d* = 0.0006). However, the decrease in mortality rates following ischemic stroke over the period was statistically significant for both sexes (Table 3). In men, the raw mortality rate fell from 10.6 % in 2008 to 9.8 % in 2011 (*Somers’d* < 10^−3^) and in women it fell from 13.4 % in 2008 to 12.7 % in 2011 (*Somers’d* = 0.038). This decrease concerned all age groups for men and the more than 55 years group for women.

We found no significant variation in mortality rates according to age group and sex for hemorrhagic strokes (Table 2).

Table 4 shows that, in our population, the risk of dying in hospital in the 30 days following a stroke whatever the type was lower in men than in women (relative risk: male/female (RR): 0.86 [95 % CI 0.84–0.87]). Nonetheless, the RR was different depending on the age group and type of stroke. For both types (hemorrhagic and ischemic), women older than 55 years presented a higher risk of death than men of the same age group (RR: 0.97 [95 % CI 0.95–0.98] for hemorrhagic and RR: 0.78 [95 % CI 0.77–0.80] for ischemic). However, for patients younger than 36 years, the risk was lower in women for both types of stroke (RR: 1.23 [95 % CI 1.02–1.48] for hemorrhagic and RR: 1.68 [95 % CI 1.11–2.53] for ischemic), and for hemorrhagic stroke in women aged 36–55 years (RR: 1.15 [95 % CI 1.08–1.23]).

**Table 4:** Relative risk of in-hospital mortality, comparison between males and females

	Relative risk (RR:male:female)								
	Hemorrhagic + ischemic strokes			Hemorrhagic strokes			Ischemic strokes		
	RR	95 % CI		RR	95 % CI		RR	95 % CI	
Age									
≤35	1.51	1.28	1.80	1.23	1.02	1.48	1.68	1.11	2.53
36–55	0.97	0.91	1.03	1.15	1.08	1.23	1.12	0.98	1.27
>55	0.87	0.86	0.88	0.97	0.95	0.99	0.81	0.79	0.82
All	0.86	0.84	0.87	0.97	0.95	0.98	0.78	0.77	0.80

*CI* confidence interval

### Spatial analysis of in-hospital mortality

**Fig. 3:**
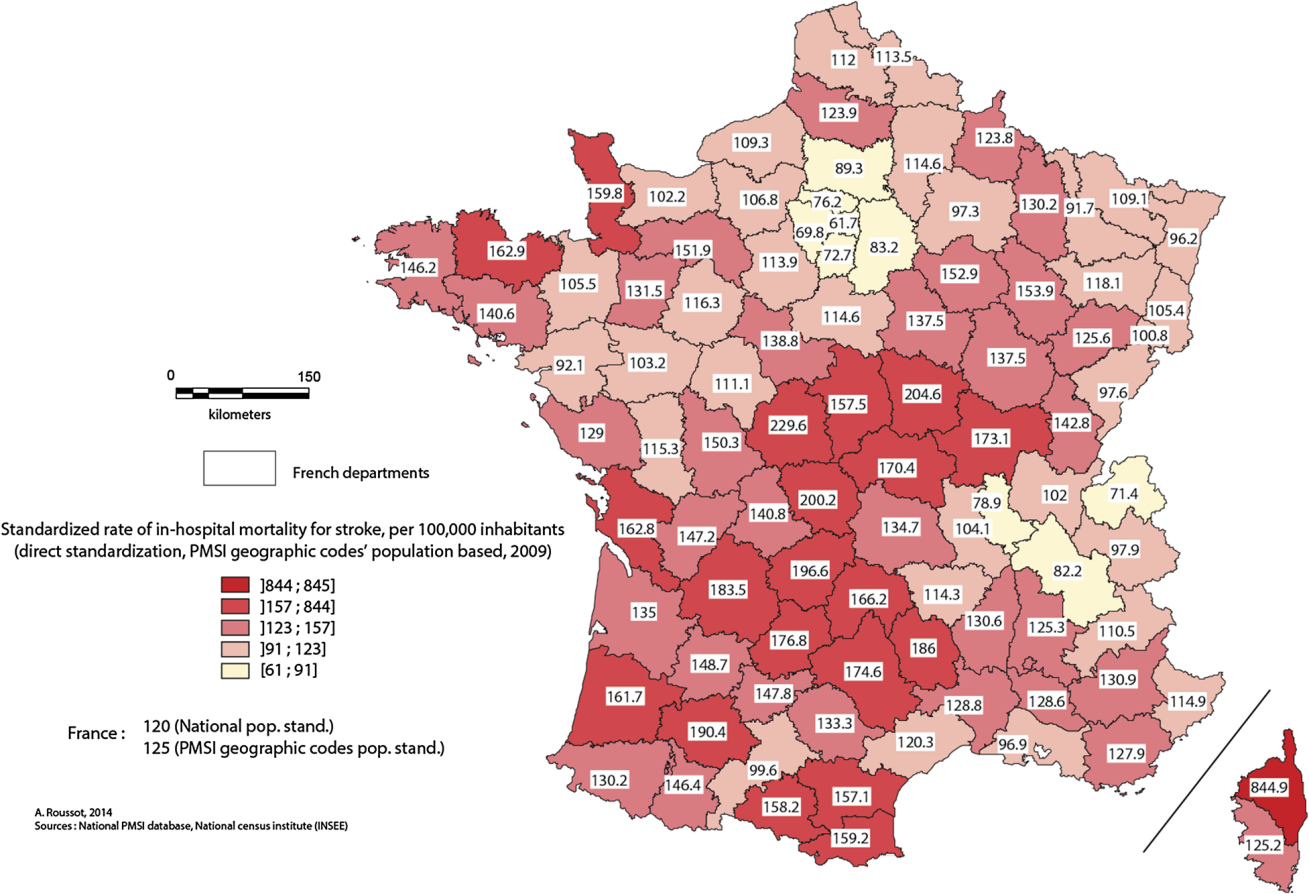
Standardized rate of in-hospital mortality at the departmental level, 2008–2011

The overall decrease in in-hospital mortality over the 4 years of the study masks stark spatial differences in France (c.f. Fig. 3). The map of the standardized rates illustrates a concentration of high stroke-related mortality along a north-east/south-west diagonal, as well as high rates in Brittany departments. The highest rates are seen in central departments and in Haute-Corse, in north of Corsica. In contrast, the departments of Ile-de-France, Rhône, Isere and Haute-Savoie have the lowest rates.

**Fig. 4:**
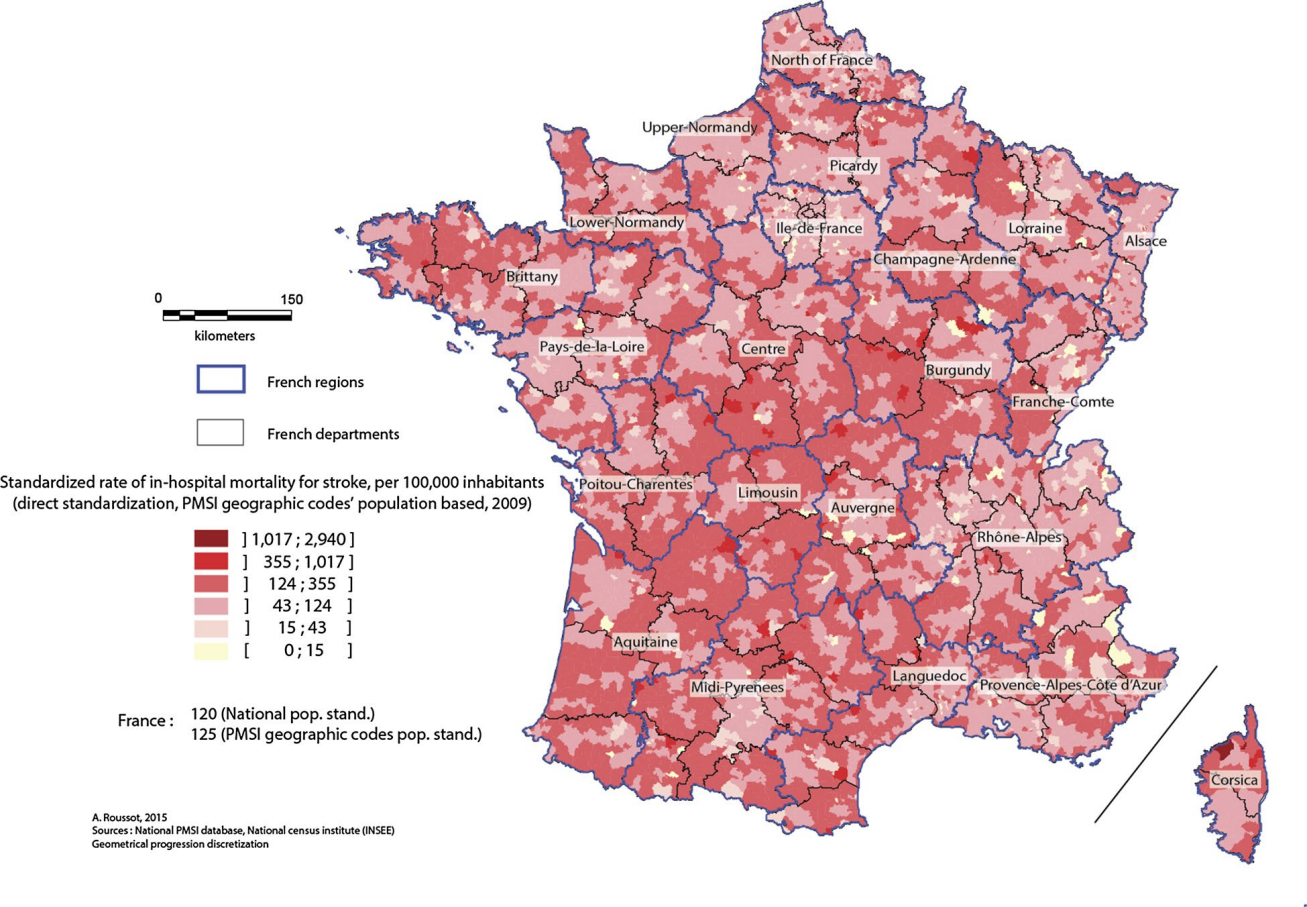
Standardized rate of in-hospital mortality at the level of PMSI geographic codes, 2008–2011

At the level of geographic codes (c.f. Fig. 4), the map shows clusters with high in-hospital mortality, notably in central Brittany and along a geographical diagonal of high mortality through the country, following a north-east/south-west axis. Areas bordering the Rhône and Loire valleys, Alsace and the north-east of the Rhone-Alps region, however, appear to be better protected. In Corsica, areas around the town of Calvi, in the north-west of the island are particularly affected by high mortality.

Tables 5 and 6 present the results of the ascending hierarchical classification at the level of PMSI geographic codes and the characteristics of each cluster. Cluster 1 represents urban areas, near large towns or areas that benefit from the attraction of nearby large towns. Stroke-related mortality is low in these areas; the population is younger than elsewhere, as shown by the lower proportion of over 60 s and pensioners, but the occupational profile was more precarious, with a high proportion of unemployed, intermediate workers and manual workers compared with the low proportion of managers or professional occupations.

**Table 5:** Results of the HAC

Variable	Overall mean	Cluster 1	Cluster 2	Cluster 3	Cluster 4	Cluster 5
		Prob (test) > 0	Prob (test) > 0	Prob (test) > 0	Prob (test) > 0	Prob (test) > 0
Farmers (%)	3.30	<0.0001	<0.0001	<0.0001	<0.0001	<0.0001
Craftsmen–storekeepers—entrepreneurs (%)	6.91					
Managers or professional occupations (%)	11.20				0.2305	
Intermediate professions—technicians (%)	22.99				0.0244	
Employees (%)	28.48					
Skilled workers (%)	26.15				<0.0001	
0–20 years old (%)	24.20					
20–60 years old (%)	51.48					
60+ (%)	24.80					
Unemployed persons (%)	10.93					
15–64 years old retired or pre-retired persons (%)	14.84					
Standardized rate of in-hospital mortality for stroke	122.58

**Table 6:** Characteristics of clusters from the HAC

Cluster	1	2	3	4	5
Socio-professional category (mean in each cluster)					
Farmers (%)	1.17	0.57	4.06	1.96	11.02
Craftsmen storekeepers entrepreneurs (%)	5.49	6.26	6.23	9.68	9.39
Managers or professional occupations (%)	9.96	24.65	8.54	NS	6.56
Intermediate professions—technicians (%)	23.41	29.15	21.95	23.32	17.77
Employees (%)	30.96	24.24	26.86	31.53	26.97
Skilled workers (%)	27.69	14.38	31.61	21.32	27.29
Demographic structure (mean in each cluster)					
0–20 years old (%)	25.47	26.24	25.77	20.69	19.45
20–60 years old (%)	53.05	53.91	52.25	48.42	46.95
60+ (%)	21.90	20.07	22.53	31.36	34.30
Sanitary variable (mean in each cluster)					
Unemployed persons (%)	13.15	7.80	9.30	12.94	10.12
15–64 years old retired or pre-retired persons (%)	13.50	11.33	13.77	18.63	19.75
Standardized rate of in-hospital mortality for stroke (per 100,000 inhabitants)	105.13	76.47	114.07	155.41	193.41

Cluster 2, which also represents urban and peri-urban France, has low stroke-related mortality but unlike cluster 1 has a high socio-economic status with a higher proportion of managers and professional occupations and a lower proportion of manual workers and unemployed than in other clusters. This cluster mainly includes peri-urban areas situated in the affluent suburbs of large agglomerations: west of Ile-de-France, Lille, Nancy, Strasbourg, Lyon, Montpellier, Toulouse, Bordeaux, Nantes, Rennes.

Cluster 3 includes dynamic rural areas of the west and east of France. Stroke-related mortality in these areas is relatively low and the population is relatively younger than in other clusters.

The areas in cluster 4 are mostly situated along the coast in the south and south-east of the country in Provence, in the Alps, and Corsica, and are characterized by an aging population, high levels of unemployment and high stroke-related in-hospital mortality.

**Fig. 5:**
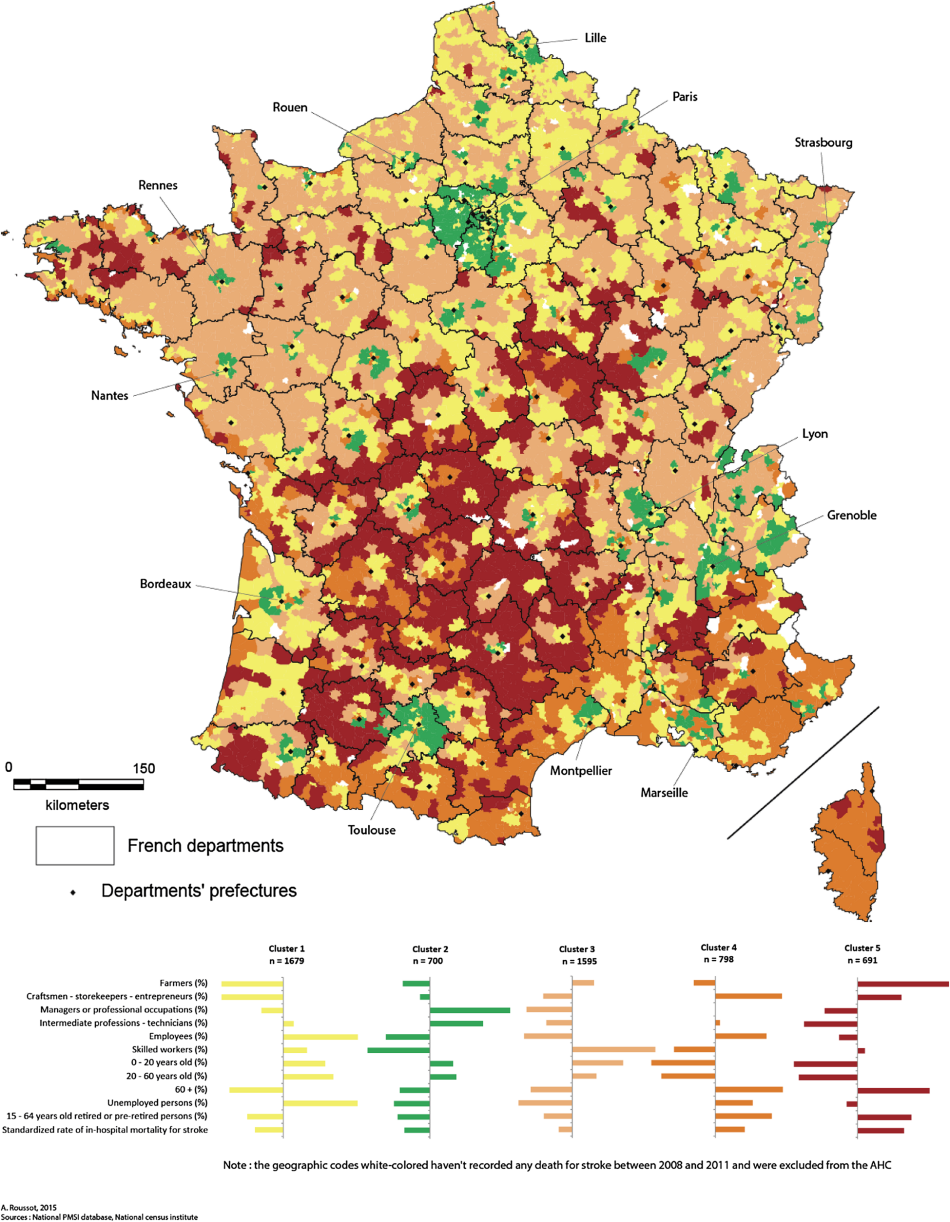
Cartography of the results of the ascending hierarchic classification

The spatial distribution of high stroke-related in-hospital mortality seems to follow the north-east/south-west axis (cf. Figs. 3, 4), as shown by the results of the AHC (cf. Fig. 5). Areas with low in-hospital mortality are also those with favorable socio-professional and demographic structures. Areas with high mortality (Fig. 4) corresponded visually with areas in cluster 5, which have low-income occupations, pensioners or pre-retirees and an older population.

For the country as a whole, the spatial distribution of socio-economic and healthcare indicators seems to favor areas around large towns or departmental prefectures. Young people and tertiary-sector professions are concentrated in these main towns (cluster 1), which have a denser healthcare structure and show low stroke-related mortality. The affluent suburbs of these main towns (cluster 2) are organized in a concentric pattern around these urban centers. Further away, rural areas associated with these urban centers (cluster 3) also have low mortality, but also include large underprivileged areas with aging populations (clusters 4 and 5).

## Discussion

The list of diagnostic codes used to select patients corresponded to those used in other studies on stroke mortality, in France [8], and internationally [9]. Different sensitivity and validation analyses have shown the robustness of the methodology [10, 11]. However, following a validation study of PMSI data [8], our selection criteria differed slightly from those of Peretti et al. Our selection of hemorrhagic stroke was based on codes I60 and I61 and notably on code I62.9. We did not use all I62 codes, given their lower sensitivity according to a validation study of PMSI data [8]. Moreover, patients suffering from TIA were not included in our study given the heterogeneous nature of coding practices [12].

According to the national PMSI databases, overall, the number of patients hospitalized for stroke increased in France from 2008 to 2011, despite a fall between 2010 and 2011. This increase was not homogeneous and depended on the age group. The greatest increases affected patients older than 55 years, which is in line with demographic change: in France according to the INSEE, the number of persons aged over 55 years increased by 2.5 % between 2008 and 2011.

The majority of patients hospitalized following stroke were women, which is in keeping with the results of an earlier study conducted in France [13]. Moreover, our study shows that the increase in the proportion of patients older than 55 years affects women more than men in this his age group. This result agrees with the results of recent study [14], which show that with the increased life expectancy in developed countries, women contribute more than men to the increased prevalence and incidence of stroke in the oldest age group. This increased life expectancy without stroke was shown in an earlier study, which reported a mean increase of 5 years in men and of 8 years in women of stroke in Dijon, France between 1987 and 2008 [15]. This trend over the same period was also found in Sweden [16] and in New-Zealand [17].

The increase concerning the 36–55 years age group for ischemic stroke, notably in men, is in keeping with recent data, whether from different population registries or from medico-administrative data [7], and with trends observed worldwide [1, 18, 19]. This increase is certainly multifactorial and raises the problem of uncontrolled or increasing risk factors in this population. Such factors include smoking, diabetes, hypercholesterolemia, obesity, or cannabis consumption [18, 20–25]. These results indicate that primary vascular prevention is necessary throughout life and should start in childhood.

At the same time, the characteristics of the hospital stay have changed. First, the mean length of hospital stay decreased and secondly, there was a clear fall in in-hospital mortality. According to a validation study conducted by the Technique de l’Information Hospitaliere (ATIH) and INSEE, the quality of recording in-hospital mortality in the PMSI database has improved. We thus focused a part of our analysis on this indicator of performance of the healthcare system, especially since this type of analysis is becoming more widespread with the growing use of medico-administrative data [26–28]. The reduced in-hospital mortality observed for hospital stays overall corresponds to observations of a French study, which reported that the proportion of intra-hospital deaths during the first hospital stay for stroke had fallen significantly by 0.5 percentage points between 2007 and 2009 [7]. These observations could be related to the better reactivity of hospital emergency departments and the earlier arrival of patients. On this subject, another study pointed out the efficacy of fibrinolysis in patients with ischemic stroke in a context of reinforced on-call duty in specialized neurology units, stroke units [29, 30].

Our results also indicate that raw and standardized mortality rates were significantly higher in women than in men, which was also shown in another study conducted in France [13]. However, this excess mortality following stroke in women was not found at the level of all developed countries, where mortality rates in men are higher than those in women [31]. In our study, however, this excess mortality in women was only found for women aged 56 years and over. Yet, for this age group, the mean age of women was 80 and 5 years older than that of men. As mortality increases with age, this difference in mean ages may explain the excess mortality. In contrast, there was excess mortality in men younger than 56 years for hemorrhagic stroke, and in those younger than 36 years for ischemic stroke. The RR observed in our study for these age groups were of the same order of magnitude as those observed in the United States in young adults [32].

Our spatial study is the first to be conducted at the level of PMSI geographic codes in France. The results using this approach were more accurate than those obtained at the department level. Indeed, earlier studies have already investigated the geographic distribution of stroke prevalence and mortality at the global scale [1, 33]. These studies showed an increase in stroke prevalence associated with a fall in mortality in high-income developed countries, like France. However, we do not have such a small-scale spatial analysis of stroke-related mortality in France. Our work therefore showed the interest of associating medical data with socio-economic data from the national census, which is available at the post-code level. Concerning in-hospital mortality, this association could be used for other diseases and in other countries, depending on the availability of databases and the scale of analysis they allow.

Two major results came out of this study. The map of standardized mortality rates allowed us first of all to illustrate the territorial disparities at the national level, and notably by identifying areas of high mortality from the north-east to the south-west of the country, along the “low population density diagonal”. Areas along this diagonal are characterized by aging communities, but the excess mortality cannot be explained by age differences alone as age-standardized mortality was also higher than elsewhere. This zone, which we call the “excess mortality diagonal” had already been pointed out by French geographers, notably in terms of premature death in the population at large [34]. Another hypothesis to explain the high mortality rate could be that these territories are often far from emergency care facilities; this aspect has already been mentioned in several studies [35, 36]. Moreover, if we consider that patients hospitalized for stroke are hospitalized as close as possible to their homes, given that emergency care facilities are supposed to be nearby, these results indicate the location of “protective” and “accidentogenic” zones on a very small scale. Local observations could provide a diagnostic tool for decision-makers to assess the quality of prevention, to evaluate the efficacy of public healthcare messages and the burden of risk factors in certain areas [37, 38].

The isolation of certain rural areas raises the question of the development of telemedicine in the most serious cases of stroke, which require fibrinolysis. Concerning the management of ischemic strokes, telemedicine makes it possible to link small isolated rural hospitals with major hospitals where neurologists are available round the clock. Teleconsultations allow duty neurologists to help doctors in smaller hospitals to perform thrombolysis when necessary. The deployment of such systems enables the earlier management of stroke victims close to where the stroke occurred and avoids transfers to distant hospitals or stroke units. Telemedicine programs are being deployed in several French regions, notably in Burgundy, a region marked by the isolated rural areas in the centre and recent hospital restructuration. A previous study in the region brought to light the link between the isolation of populations from the healthcare system and poorer results in perinatal care [39]. However, an overall evaluation of these telemedicine programs using national claims data is difficult to achieve, as they are often too recent and because the coding of telemedicine procedures is not consistent. Studies to identify patients who have benefitted from telemedicine are nonetheless under way, but researchers need to go back to patients’ medical records at the various establishments [40, 41]. Moreover, several tests were conducted with different variables in the AHC for this study, but we chose to represent the analysis that generated the greatest inertia (69.2 %). The results obtained with variables relative to access to the healthcare system were less satisfactory: these variables were time to reach a stroke unit or a hospital emergency unit, in minutes for each geographic code.

Standardization of mortality rates on a small scale, according to the geographic code of the patient’s place of residence, revealed local clusters of high mortality, notably in Brittany. This analysis of local situations, which is more detailed than analyses at the departmental level, highlighted territorial disparities, but cannot replace the results of even smaller scale analyses, at the level INSEE codes for communes, for example. We intend to continue our work using SNIIRAM data, which will allow us to break away from the use of aggregated population data to calculate incidences or comparative indices of mortality. An earlier study underlined the interest of analyzing stroke incidence at the IRIS level (Ilots regroupés pour l’information statistique) using registry data [42, 43]. The analysis established a link between an underprivileged environment and the greater incidence of stroke. Mortality rates were also higher among lower wage earners.

The 5 clusters from the classification divide the territory according to the weight of each variable selected by the AHC. Classification was preferred for this study because it can be used to establish a solid typology that maximizes the resemblance between observations (PMSI geographic codes) within the same cluster. Concerning the ageing population in certain areas, the model presented includes the demographic structure of the geographic codes of the place of residence, as well as the smoothed rate of in-hospital mortality. These variables are treated independently in the classification, which allowed us to contextualize certain high mortality rates. Despite the adjustment for age, areas marked by high mortality were also those with the oldest population. The classification showed that the demographic structure was not the only factor of the socio-territorial organization to affect mortality. Indeed, apart from the age of the population, disparities in mortality also followed the territorial distribution of socio-professional categories. It thus appears that social difficulties and problems linked to the ageing of the population are associated with higher stroke-related mortality than is the case in certain rural or peri-urban areas. The interest of the AHC is justified by the difficulty of linking the healthcare results of a large population with their socio-economic conditions as there are as yet no indicators of this type at the individual level in French medico-administrative databases. Clustering techniques and the geographic approach provided an idea of aggregated healthcare results at a territorial level and illustrated the interplay between the socio-economic environment and healthcare issues, such as, among others, mortality in England and Wales [2] or access to the healthcare system in France [44, 45].

## Conclusions

Since the implementation of the National Stroke Plan, most studies have reported better management of patients in France, where the incidence of stroke was amongst the lowest in the world, thanks to the geographic, social and healthcare conditions in the country [46]. In this respect, in-hospital mortality in patients with ischemic stroke has markedly improved thanks to the reinforcement of the stroke units network and the generalization of fibrinolysis and in spite of the increase in the number of people hospitalized. This study makes it easier to understand that the fall in in-hospital mortality was not uniform throughout the country and was accompanied by considerable territorial diversity. We showed that the spatial distribution of healthcare indicator such like in-hospital mortality follows the distribution of demographic and social inequalities. The clustering method showed that areas characterized by unfavorable socio-economic indicators are also affected by high in-hospital mortality. We also know that areas on the “excess mortality diagonal” are often far away from the nearest emergency care facilities. Our study was conducted in the context of a wider program of the territorial organization of healthcare policies to counter the isolation and aging of rural areas. The development of telemedicine programs, another priority axis of the National Stroke Plan, should accelerate the remote management of patients and guarantee better integrated healthcare in these isolated communities.

### Ethics approval and consent to participate

This study was approved by the National Committee for data protection (Commission Nationale de l’Informatique et des Libertés, registration number 1576793) and was conducted in accordance with French legislation. The PMSI database was transmitted by the national agency for the management of hospitalization data (ATIH number 2015-111111-47-33).

## Consent for publication

Written consent was not needed for this study.

## Abbreviations

PMSI-MCO: Programme de médicalisation des systèmes d’information en Médecine, Chirurgie et Obstétrique
SNIIR-AM: Système national inter-régime de l’assurance maladie
ICD 10: International Classification of Diseases, 10th revision
PD: principal diagnosis
DRG: Diagnosis Related Group
INSEE: Institut National de la Statistique et des Etudes Economiques
ATIH: Agence Technique de l’Information Hospitalière
PCA: principal components analysis
AHC: ascending hierarchical classification
GIS: geographic information system
LOS: length of hospital stay
